# One Step beyond Species Description: Unveiling a Fine-Scale Diversity within the Genus *Dzhanokmenia* Kostjukov (Hymenoptera: Eulophidae) †

**DOI:** 10.3390/insects15060406

**Published:** 2024-06-01

**Authors:** Bolormaa Ganbaatar, Qin Li, Ouyan Xi, Huanxi Cao, Chaodong Zhu

**Affiliations:** 1 Key Laboratory of Zoological Systematics and Evolution, Institute of Zoology, Chinese Academy of Sciences, Beijing 100101, China; bolor88maa@gmail.com (B.G.); zhucd@ioz.ac.cn (C.Z.); 2 College of Life Sciences, University of Chinese Academy of Sciences, Beijing 101408, China; 3 College of Life Science and Technology, Xinjiang University, Urumqi 830046, China; liqptero@163.com (Q.L.); xoy@stu.xju.edu.cn (O.X.); 4 Xinjiang Key Laboratory of Biological Resources and Genetic Engineering, Urumqi 830046, China; 5 National Animal Collection Resource Center, Institute of Zoology, Chinese Academy of Sciences, Beijing 100101, China

**Keywords:** chalcids, taxonomy, DNA barcoding, species delimitation, intra- and interspecific variation

## Abstract

**Simple Summary:**

Chalcidoidea (chalcid wasps) are one of the most numerically, structurally, and biologically diverse groups in Hymenoptera. However, the conserved morphology coupled with a high intraspecific variability in certain key characters hinders research on chalcids. In this study, DNA barcoding with the COI gene was used to calibrate intra- and interspecific variation in diagnostic characters within the genus *Dzhanokmenia*. In addition, the nuclear gene, 28S D2, was used to infer a phylogeny to better understand the definition of *Dzhanokmenia* and its relationships to potentially close genera associated with *Tetrastichus*. A DNA barcode library that includes eight species was established for *Dzhanokmenia*. A new species, *D*. *brevifunis* Ganbaatar & Cao sp. nov., was described and illustrated. The results show that *Dzhanokmenia* is closely related to *Baryscapus* and suggest that the species diversity of *Dzhanokmenia* is underestimated. This study lays a foundation for further research on the diversity and classification of *Dzhanokmenia*.

**Abstract:**

Although Chalcidoidea is one of the megadiverse superfamilies in Hymenoptera, numerous species are still being discovered and described. However, the difficulties in delimiting intra- and interspecific variation hinder this process. In this study, DNA barcoding methods using the COI gene were employed to investigate the morphological variation within *Dzhanokmenia* Kostjukov, 1977. The nuclear locus, 28S D2, was used to infer a phylogeny to gain an understanding of the relationship of *Dzhanokmenia* with other potentially close genera. Through a preliminary DNA barcode library established here, including eight species, we calibrated the intraspecific variation in certain diagnostic characters for the new species described here, *D*. *brevifunis* Ganbaatar & Cao sp. nov. Maximum likelihood results show that *Dzhanokmenia* is clustered with the genera associated with *Tetrastichus*, such as *Chaenotetrastichus* Graham, 1987, *Baryscapus* Förster, 1856, *Tetrastichus* Haliday, 1844, and *Oomyzus* Rondani, 1870 involved in this study. Our results indicate that the species diversity of *Dzhanokmenia* is understudied and tentatively confirm that *Dzhanokmenia* has a potential close relationship with *Baryscapus*. Along with the DNA barcode library, the referenced phylogeny datasets improve the understanding of the systematic position of *Dzhanokmenia* within the subfamily Tetrastichinae and the definition of this genus in terms of morphology, thereby facilitating species delimitation, discovery, and description within *Dzhanokmenia*.

## 1. Introduction

Chalcidoidea, commonly known as chalcid wasps, represent one of the most diverse superfamilies within Hymenoptera, encompassing high species abundance and biological diversity. More than 22,000 species have been documented in Chalcidoidea, yet the total species count is estimated to surpass 500,000 [1]. Currently, Eulophidae are the largest family in terms of species diversity, comprising over 6000 valid species. Eulophidae are typically divided into four subfamilies, as confirmed by molecular data in Gauthier et al. [2]: Entedoninae, Entiinae (formerly known as Euderinae), Eulophinae, and Tetrastichinae. In 2011, the Opheliminae, which were previously recognized as a tribe within the Entedoninae, were elevated to subfamily status in a phylogenetic analysis that integrated molecular and morphological data [3]. This classification has been further supported by subsequent molecular studies [4,5]. Of the five subfamilies, the Tetrastichinae is the largest subfamily in terms of both the number of genera and species, with over 100 genera and more than 2000 species [1,6]. Two prominent genera in particular, *Aprostocetus* Westwood, 1833 and *Tetrastichus*, account for more than half of the total number of species within this subfamily [1].

*Dzhanokmenia* is one of nineteen genera within the subfamily Tetrastichinae that contains more than ten species. Until this study, a total of fifteen described species were known for this genus, all of which originated from Asia. Based on morphology and trophic associations, Kostjukov [7] established and treated *Dzhanokmenia* as one of seventeen subgenera within the genus *Tetrastichus* and designated *Tetrastichus bibikovae* Dzhanokmen, 1971 as the type species for *Dzhanokmenia*. Later, Kostjukov [8,9] described an additional six species and provided a key to the seven species of this subgenus. In a taxonomic revision of the European genera of Tetrastichinae, Graham [10] elevated *Dzhanokmenia* to generic status and considered this genus to be closely related to *Baryscapus*, especially the *evonymellae* group of *Baryscapus*, to which most species of this genus belong.

Eight further species were subsequently described within the genus. One was documented from Turkmenistan [11], two were found in the Asiatic region of Russia [12,13], and the remaining five were described from China [14,15]. To date, all fifteen known species of *Dzhanokmenia* originate from the arid and semi-arid regions of Asia, including China, Kazakhstan, southern Russia, and Turkmenistan. In addition to the five species described from China, two species, *D. demakovi* (Kostjukov, 1978) and *D. nikolskajae* (Kostjukov, 1984), have been reported from Xinjiang, China [16]. The host information for *Dzhanokmenia* has long remained unknown until recently, when *D. stefaniolae* Li, Wang & Hu, 2021, was documented to emerge from stem galls induced by *Stefaniola* sp. (Diptera: Cecidomyiidae) on *Haloxylon ammodendron* (C. A. Mey, 1829) (Chenopodiaceae) [15]. This discovery suggests that *Dzhanokmenia* species are associated with gall-forming insects inhabiting *Haloxylon* spp. (Tamaricaceae) and potentially with insects inhabiting *Tamarix* spp. (Tamaricaceae) as well [14,15].

Although considerable progress has been made in the systematics of the Chalcidoidea [3,4,5,17,18,19,20,21], fundamental taxonomic tasks such as species inventories, identifications, and descriptions remain essential for a comprehensive understanding of the biodiversity of Chalcidoidea [22,23,24,25]. Despite these taxonomic advances in the study of *Dzhanokmenia* research, the species diversity within this genus may be underestimated, given the wide distribution of its insect and plant hosts, particularly in arid and semi-arid regions. Limited knowledge of the relationships between *Dzhanokmenia* species and of intra- and interspecific variation hinder species discoveries in this genus. Furthermore, despite its elevation from a subgenus of *Tetrastichus* to a valid genus, much remains unknown about the relationships between *Dzhanokmenia* and genera that may be closely related to *Tetrastichus*.

One of the objectives of this study is to describe a new species of *Dzhanokmenia* collected in Qinghai and Xinjiang, China, and another objective is to investigate the phylogeny of *Dzhanokmenia* and closely related genera. In this study, the COI barcodes were generated to investigate intra- and interspecific variation within *Dzhanokmenia*. Additionally, the 28S D2 gene of eight *Dzhanokmenia* species recorded from China was used to preliminarily discuss the relationship of *Dzhanokmenia* with other genera associated with *Tetrastichus*, by combining a filtered DNA matrix provided by Cao et al. [25]. Our DNA barcoding analysis revealed an additional species closely allied to *D. brevifunis*, suggesting that the species diversity, or at least the genetic diversity, of *Dzhanokmenia* may not yet be adequately explored. Phylogenetic analysis based on the 28S D2 gene confirmed the hypothesis that *Dzhanokmenia* may be closely related to *Baryscapus*.

## 2. Materials and Methods

### 2.1. Samples Collection

The specimens of the new species were collected in 2009, 2013, and 2017 in Xinjiang and Qinghai, China, using sweep nets and Malaise traps. Upon arrival at the laboratory in the Institute of Zoology, Chinese Academy of Sciences (IOZCAS), the Eulophidae specimens were separated from the bulk insect samples and preserved in 95–100% ethanol for subsequent analyses. The specimens of *Dzhanokmenia karamayica* Li, Wang & Zhu, 2016, and *D. gobica* Li, Wang & Zhu, 2016, were collected by sweeping herbaceous plants or shrubs in the semi-arid regions of Xinjiang, China, as documented in Li et al. [13]. Subsequently, the *Dzhanokmenia* specimens were sorted out in the laboratory at Xinjiang University and preserved in 95–100% ethanol before being transferred to the laboratory at IOZCAS for subsequent sequencing.

### 2.2. Specimen Preparations

The specimens used for morphological studies were dried with a Leica EM CPD300 automated critical point dryer (Leica Microsystems, Wetzlar, Germany). Subsequently, some specimens were mounted on cards, while others were dissected into head, mesosoma, and metasoma to obtain micrographs using a scanning electronic microscope (SEM). Specimens were examined using a Nikon SMZ 1500 stereomicroscope equipped with a 10 mm ocular grid with 100 divisions. The habitus images were taken with a Nikon D7000 digital camera connected to the stereomicroscope. The prepared parts used for the SEM were coated with gold using a Leica EM SCD050 super cool sputter coater. Micrographs were taken with an FEI Quanta 450 environmental scanning electron microscope. All color images were stacked using Helicon Focus software 8.2.2.

The terminology follows Gibson [26], except for substituting ”scutellum” with “mesoscutellum”. Abbreviations are as follows: F1–F3, funiculars 1–3; Gtn, gastral tergite number; MLM, midlobe of mesoscutum; MV, marginal vein; OOL, the shortest distance between an eye and a posterior ocellus; POL, the shortest distance between the posterior ocelli; SMV, submarginal vein. The type specimens of the new species described in this study are deposited in the National Animal Collection Resource Center of IOZCAS.

### 2.3. DNA Extraction, Amplification and Sequence Editing

Fifty-two specimens of *Dzhanokmenia* from Qinghai and Xinjiang, China, were processed for whole-genomic DNA extraction using the DNeasy Blood & Tissue Kit (Qiagen GmbH, Hilden, Germany), following the manufacturer’s instructions (see successful sequencing specimens in Table 1). The specimens of *Dzhanokmenia* selected for the DNA extraction of the new species were chosen to capture all observed variation. To elucidate the phylogenetic relationship between *Dzhanokmenia* and *Baryscapus*, a representative species of *Baryscapus* was also chosen for DNA extraction. Subsequently, the barcoding region of the COI gene was generated for all specimens. Additionally, a specimen of *Tetrastichus howardi* (Olliff, 1893) was included in the amplification of the COI gene. Thirty-six specimens representing all analyzed species of *Dzhanokmenia* were selected to generate the 28S D2 region for phylogenetic analyses. All primers used in this study are listed in Table 2. The PCR reactions for COI were performed by following the protocol described by Huangfu et al. [27], while the reactions for 28S D2 were based on the protocol established by Cao et al. [25].

The raw sequences were assembled, edited, and aligned using BioEdit version 7.0.9.0 [28]. The COI matrix was translated into amino acids in MEGA7.0 [29] to identify any stop codons. In addition to these newly generated sequences, we downloaded publicly available sequences from GenBank (Table S1), as generated or utilized in Cao et al. [25]. Three DNA matrices were generated for subsequent maximum likelihood (ML) analyses. First, the generated 28S sequences were combined and aligned with a filtered matrix of 28S sequences provided by Cao et al. [25]. Identical sequences were then merged into one to form MatrixI. Second, based on the ML tree on MatrixI (Figure S1), MatrixII was refined from MatrixI to eliminate distant sequences from *Dzhanokmenia* according to the ML result of MatrixI, while retaining *Styotrichia pisoxylona* Cao & Zhu, 2024 as an outgroup. Lastly, the aligned COI and 28S sequences for specimens that had been successfully sequenced for both genes were concatenated, resulting in MatrixIII, which enables subsequent phylogenetic analyses based on two genes.

The voucher specimens used to generate the molecular data in this study have been deposited at IOZCAS. The DNA sequences obtained in this study have been submitted to GenBase, and the accession numbers can be found in Table 1. In addition, the information of other publicly available sequences used in the above three matrices has been provided in Table S1.

**Table 1:** List of specimens used in molecular analyses, with GenBase accession numbers.

Voucher Number	Species	Sex	Locality	COI	28S
CHX_DZH_117	*D. brevifunis*	female	Xinjiang, Aletai	C_AA071359.1	C_AA071403.1
CHX_DZH_118	*D.* cf. *brevifunis*	female	Xinjiang, Aletai	C_AA071360.1	C_AA071404.1
CHX_DZH_119	*D. brevifunis*	female	Xinjiang, Aletai	C_AA071361.1	C_AA071405.1
CHX_BAR_177	*Baryscapus* sp.	female	Beijing	C_AA071358.1	C_AA071402.1
CHX_DZH_737	*D. brevifunis*	female	Qinghai, Ge-Ermu	C_AA071362.1	C_AA071406.1
CHX_DZH_738	*D. brevifunis*	female	Qinghai, Ge-Ermu	C_AA071363.1	C_AA071407.1
CHX_DZH_739	*D. brevifunis*	female	Qinghai, Ge-Ermu	C_AA071364.1	C_AA071408.1
CHX_DZH_740	*D. brevifunis*	female	Qinghai, Ge-Ermu	C_AA071365.1	C_AA071409.1
CHX_DZH_741	*D. brevifunis*	female	Qinghai, Ge-Ermu	C_AA071366.1	C_AA071410.1
CHX_DZH_742	*D. brevifunis*	female	Qinghai, Ge-Ermu	C_AA071367.1	C_AA071411.1
CHX_DZH_743	*D. brevifunis*	female	Xinjiang, Tulufan	C_AA071368.1	C_AA071412.1
CHX_DZH_744	*D. brevifunis*	female	Xinjiang, Tulufan	C_AA071369.1	C_AA071413.1
CHX_DZH_747	*D. brevifunis*	female	Xinjiang, Tulufan	C_AA071370.1	C_AA071414.1
CHX_DZH_748	*D. brevifunis*	female	Xinjiang, Tulufan	C_AA071371.1	C_AA071415.1
CHX_DZH_888	*D.* cf. *brevifunis*	female	Xinjiang, Aletai	C_AA071372.1	C_AA071416.1
CHX_DZH_890	*D. brevifunis*	female	Xinjiang, Aletai	C_AA071373.1	-
CHX_DZH_891	*D.* cf. *brevifunis*	female	Xinjiang, Aletai	C_AA071374.1	C_AA071417.1
CHX_DZH_892	*D. brevifunis*	female	Xinjiang, Tulufan	C_AA071375.1	-
CHX_DZH_893	*D.* sp.1	female	Xinjiang, Tulufan	C_AA071376.1	-
CHX_DZH_894	*D.* cf. *brevifunis*	female	Xinjiang, Tulufan	C_AA071377.1	-
CHX_DZH_895	*D. brevifunis*	female	Xinjiang, Tulufan	C_AA071378.1	-
CHX_DZH_896	*D.* cf. *antonovae*	female	Xinjiang, Aletai	C_AA071379.1	C_AA071418.1
CHX_DZH_897	*D.* cf. *antonovae*	male	Xinjiang, Aletai	C_AA071380.1	C_AA071419.1
CHX_DZH_898	*D.* sp.2	female	Xinjiang Aletai	C_AA071381.1	C_AA071420.1
CHX_DZH_900	*D. brevifunis*	female	Xinjiang, Tulufan	C_AA071382.1	-
CHX_DZH_901	*D. brevifunis*	female	Qinghai, Germu	C_AA071383.1	-
CHX_DZH_902	*D.* cf. *brevifunis*	female	Xinjiang, Aletai	C_AA071384.1	C_AA071421.1
CHX_DZH_903	*D. brevifunis*	male	Xinjiang, Tulufan	C_AA071385.1	C_AA071422.1
CHX_DZH_904	*D. brevifunis*	male	Xinjiang, Tulufan	C_AA071386.1	C_AA071423.1
CHX_DZH_906	*D. karamayica*	female	Xinjiang, Karamay	C_AA071387.1	C_AA071424.1
CHX_DZH_907	*D. gobica*	female	Xinjiang, Shihezi	C_AA071388.1	C_AA071425.1
CHX_DZH_908	*D.* cf. *sugonjaevi*	female	Xinjiang, Aletai	C_AA071389.1	C_AA071426.1
CHX_DZH_909	*D. muleica*	female	Xinjiang, Karamay	-	C_AA071427.1
CHX_DZH_910	*D. gobica*	female	Xinjiang, Fukang	C_AA071390.1	C_AA071428.1
CHX_TET_054	*Tetrastichus howardi*	female	Brazil, Sete Lagoas	C_AA071391.1	OP538682.1
NT-04	*D. karamayica*	female	Xinjiang, Karamay	C_AA071392.1	C_AA071429.1
NT-05	*D. karamayica*	female	Xinjiang, Karamay	C_AA071393.1	C_AA071430.1
NT-06	*D. karamayica*	male	Xinjiang, Karamay	C_AA071394.1	C_AA071431.1
NT-07	*D. karamayica*	male	Xinjiang, Karamay	C_AA071395.1	C_AA071432.1
NT-08	*D. gobica*	male	Xinjiang, Shihezi	C_AA071396.1	C_AA071433.1
NT-09	*D. gobica*	female	Xinjiang, Shihezi	C_AA071397.1	C_AA071434.1
NT-10	*D. gobica*	male	Xinjiang, Shihezi	C_AA071398.1	C_AA071435.1
NT-11	*D. gobica*	male	Xinjiang, Shihezi	C_AA071399.1	C_AA071436.1
NT-13	*D. gobica*	female	Xinjiang, Shihezi	C_AA071400.1	C_AA071437.1
NT-16	*D. gobica*	male	Xinjiang, Shihezi	C_AA071401.1	C_AA071438.1

The symbol “-” indicates a failed case in PCR or sequencing. The 28S sequence of “CHX_TET_054” was deposited in GenBank by Cao et al. [25].

**Table 2:** List of primers used in this study.

Gene	Primer	Sequence (5′-3′)	References
COI	LCO1490	GGTCA ACAAA TCATA AAGAT ATTGG	[30]
COI	HCOout	CCAGG TAAAA TTAAA ATATA AACTTC	[31]
COI	FWPTF1	CCTGG TTCTT TRATT GGTAA TGATC	[32]
COI	Lep-R1	TAAAC TTCTG GATGT CCAAA AAATCA	[33]
28S	D2-3549F	AGTCG TGTTG CTTGA TAGTG CAG	[34]
28S	D2-3665F	AAGAGAGAGTTCAAGAGTACGTG	[35]
28S	D2-4068R	TTGGT CCGTG TTTCA AGACG GG	[34]
28S	D3-4083R	TAGTT CACCA TCTTT CGGGT CCC	[35]

### 2.4. Species Delimitation

Initially, we compiled a comprehensive list of all the examined specimens and meticulously documented their morphological variability. We assigned all examined specimens to morphospecies based on our understanding of intra- and interspecific variation, referring to the related taxonomy work of Kostjukov [7,8,9,11], Kostjukov and Kosheleva [12,13], Graham [10], and Li et al. [14,15]. Two molecular methods for species delimitation, Automatic Barcode Gap Discovery (ABGD) [36] and the Generalized Mixed Yule-Coalescent (GMYC) model [37], representing a distance-based and a tree-based coalescent approach, respectively, were employed to delimit intra- and interspecific variation and evaluate the species boundaries of these morphospecies.

ABGD automatically partitions DNA barcode sequences based on the barcode gap using iterative model-based confidence limits for intraspecific divergence [36]. Pairwise distances of *Dzhanokmenia* COI sequences were calculated based on the Kimura 2-parameter (K2P) model [38] in MEGA7.0 [29]. In addition, a Neighbor-Joining (NJ) tree [39] was constructed based on the K2P model with 1000 bootstrap replicates to calculate support values for the branches. The resulting K2P distance matrix was then analyzed using the ABGD online tool (https://bioinfo.mnhn.fr/abi/public/abgd/abgdweb.html, accessed on 20 April 2024) for partitioning. The relative gap width was set to 0.5, while other parameters were left at their default values.

The GMYC model determines intra- and interspecific branching events in a phylogenetic tree estimated from DNA sequence data [37]. For the COI gene, we performed a GMYC analysis using the single method implemented in the ‘splits’ package [37] with R 4.3.3 [40] to infer the putative species. After removing identical sequences to avoid terminal branches with zero length, ultrametric trees were estimated in BEAST v1.10.4 [41] under an uncorrelated log-normal relaxed clock model [42], HKY [43], and Gamma substitution with all other default priors. Markov chains were run for 5 × 10^7^ generations, sampling every 5 × 10^4^ generations. Tracer v1.7.2 [44] was used to visualize and evaluate the convergence of the analyses.

### 2.5. Phylogenetic Analysis

The ML analyses were conducted using IQ-TREE version 1.6.12 [45]. ModelFinder Plus (MFP) [46] was employed in all cases to automatically select the optimal substitution model, and 1000 ultrafast bootstrap replicates were performed to assess the robustness of the results. In the ML analysis of MatrixI, which contains almost all available 28S D2 sequences of Tetrastichinae that could be aligned, species from the tribe Cirrospilini (Eulophinae) were chosen as outgroups, as suggested by Gauthier et al. [2]. *Styotrichia pisoxylona* served as an outgroup in the ML analyses of MatrixII and MatrixIII. The resulting ML trees were visualized and annotated using iTOL [47].

## 3. Results

### 3.1. Species Identification and Delimitation

In the initial morphological identification, all specimens resembling *Dzhanokmenia brevifunis* were assigned to one morphospecies, with potential intraspecific variability documented. As a result, six morphospecies of *Dzhanokmenia* were identified: *D. brevifunis* sp. nov., *D. gobica*, *D. karamayica*, *D. muleica* Li, Wang & Hu, 2016, *D.* cf. *antonovae* and *D.* cf. *sugonjaevi*.

**Figure 1:**
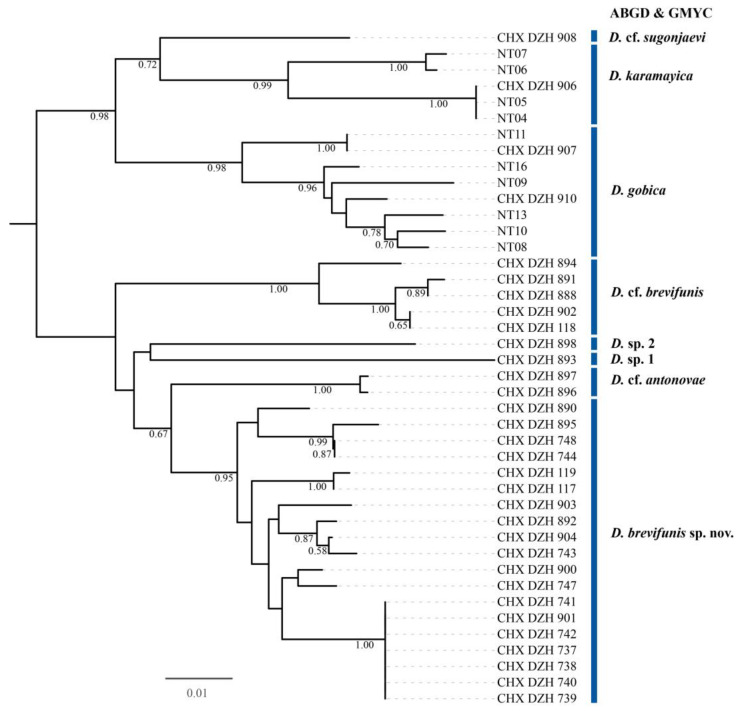
An NJ tree illustrating species delimitation results obtained using ABGD and GMYC methods based on K2P distances derived from the COI region. Bootstrap values are shown below the branches with values greater than 0.50. Each vertical bar on the right represents a distinct species delimitated by both ABGD and GMYC.

However, of the 52 individuals used for sequencing, 7 failed to yield COI sequences and were consequently excluded from the DNA barcoding analysis. The specimens used for the subsequent molecular analyses are summarized in Table 1. A total of 4 of these failed individuals belong to the species *Dzhanokmenia muleica*, resulting in its absence from the DNA barcode library. The resulting COI matrix for species delimitation comprised 42 individuals, with a sequence length of 434 bp, including aligned gaps. The results of the two molecular methods for species delimitation were consolidated in an NJ tree. Although ABGD failed to identify a barcoding gap within all available COI sequences of *Dzhanokmenia*, it suggested the presence of eight molecular units stably supported by COI sequences using this distance method. Furthermore, these eight putative species were corroborated by GMYC results. These molecular species delimitation results led to the separation of one morphospecies of *D. brevifunis* into three distinct species (see Figure 1).

### 3.2. Phylogenetic Analyses

**Figure 2:**
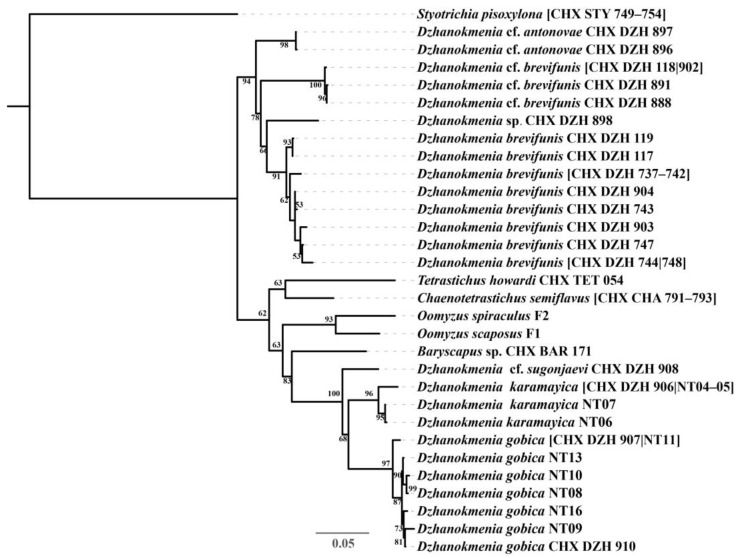
An ML tree based on MatrixIII, concatenating COI and 28S D2 regions, with *Styotrichia pixoxylona* as outgroup. Bootstrap values with values greater than 50 are shown to the left of the nodes.

DNA barcodes were successfully generated for eight *Dzhanokmenia* species recorded from China (Table 1). We expanded the 28S matrix provided by Cao et al. [24] to include 97 sequences, incorporating 35 sequences from *Dzhanokmenia* species and 1 sequence from *Baryscapus* species generated in this study. After merging identical sequences, the following three matrices were generated for the phylogenetic analyses: (1) MatrixI: comprised 72 unique sequences with a length of 429 bp (28S, including aligned gaps); (2) MatrixII: comprised 30 unique sequences with a length of 419 bp (28S, including aligned gaps); (3) MatrixIII: comprised 35 unique concatenated sequences with a length of 852 bp (COI and 28S regions, including aligned gaps). The phylogenetic trees based on these three matrices are shown in Figures S1 and S2 and Figure 2, respectively.

### 3.3. Morphological Diagnosis and Species Treatments of Dzhanokmenia

#### 3.3.1. Genus Dzhanokmenia Kostjukov, 1977

*Dzhanokmenia* Kostjukov, 1977 [7]: 189 (as subgenus of *Tetrastichus*). Type species: *Tetrastichus bibikovae* Dzhanokmen, 1971, by original designation.

*Dzhanokmenia* Kostjukov (as valid genus), Graham, 1991 [10]: 162–163.

Diagnosis. Mesosoma metallic green to blue, and metasoma with or without pale markings. Malar sulcus strongly curved. MLM with a single row of adnotaular setae on each side, exhibiting symmetrical or asymmetrical arrangement of setae. Antenna pale, although male pedicel sometimes darker dorsally at base; female funicle 3-segmented, male funicle 4-segmented. On hind leg, tarsomere 1 shortest, tarsomeres 2 and 3 subequal in length, and tarsomere 4 the longest. Fore wing except MV with extremely short marginal setae; MV and STV thickened, and SMV with one dorsal seta; hind wing knife-shaped, without marginal setae along upper margin.

Remarks. In terms of morphology, the most distinctive key character of *Dzhanokmenia* is the absence of marginal setae on the fore wing, except for MV, and the absence of marginal setae along the upper margin of the hind wing. While the MV and SMV are sometimes thickened in both the female and male, they are not as thick as those in *Dzhanokmenia*. In contrast, a key character of *Baryscapus* is the swollen scape in the male [10,48,49]. However, not all males of *Baryscapus* exhibit a swollen scape, and males are unknown in some species [48,49]. Furthermore, the genus *Oomyzus* exhibits a similar variation in male scape morphology, with some species possessing a swollen scape (*O. incertus* (Ratzeburg, 1844)) while others do not (e.g., *O. spiraculus* Song, Fei & Cao, 2020) [50]. Consequently, further studies are needed to determine whether the swollen male scape serves as a key character with phylogenetic significance. Additionally, in some *Baryscapus* species, the fore wing may also have short marginal setae on apical margin (e.g., *B. berhidanus* Erdős). We have also observed that certain species of Eulophidae (such as species of *Baryscapus* and *Neochrysocharis* Kurdjumov, 1912), Encyrtidae, Eurytomidae, and Pteromalidae, collected in semi-arid regions, possess relatively transparent fore wing discs with a reduced number of setae. This suggests that this character may be convergent due to adaptations to the environment, similar to the case of the enlarged mandibles in *Chaenotetrastichus* and *Styotrichia* LaSalle, which possibly evolved due to host adaptation [46]. While *Dzhanokmenia* species consistently have one dorsal seta on the SMV, *Baryscapus* species typically have two or more, very rarely with one seta. Therefore, the combination of characters outlined in the diagnosis of *Dzhanokmenia* is suggested to distinguish *Dzhanokmenia* species from closely related genera.

#### 3.3.2. *Dzhanokmenia brevifunis* Ganbaatar & Cao, sp. nov.

Diagnosis. Body metallic green to metallic blue green, with weak to strong bronze tinge; antenna pale yellow, basal 3/4 of pedicel slightly infuscate dorsally; legs predominantly pale yellow, fore coxa pale yellow to brown at base, mid coxa infuscate basally to entirely metallic green, hind coxa metallic green with bronze tinge. Female antenna stout, and funicle 3-segmented; F1 distinctly transverse, 1.25–1.55× as broad as long; F2 subquadrate to slightly longer than broad, 1.05–1.45× as long as broad; F3 slightly transverse to subquadrate, 0.90–1.25× as broad as long; male antenna slender and funicle 4-segmented, with F1 distinctly transverse, about 2× as broad as long, and each remaining flagellomere longer than broad.

**Figure 3:**
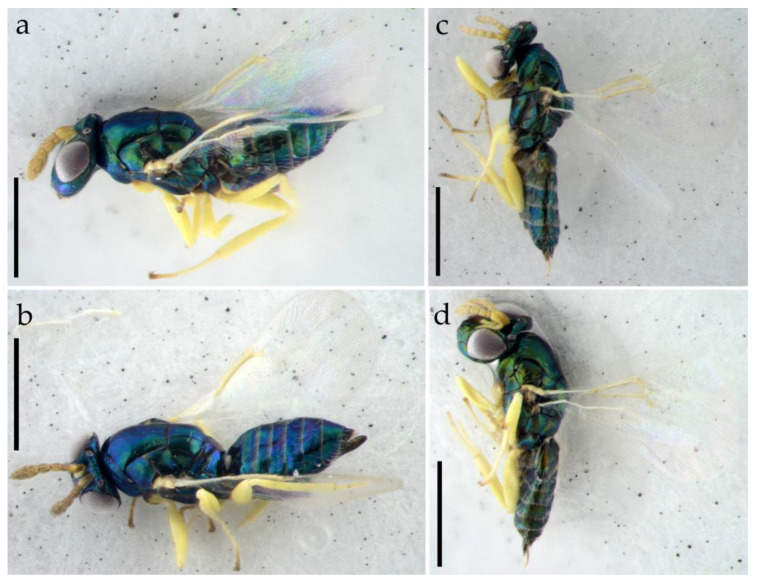
*Dzhanokmenia brevifunis* sp. nov. (**a**) Habitus of female holotype in dorsolateral view; (**b**) Habitus of female paratype in dorsolateral view; (**c**) Habitus of male paratype in lateral view; (**d**) Habitus of male paratype in dorsolateral view. Scale bar: 0.5 mm.

Female. Body length 0.9–1.5 mm. Body usually metallic green with a weak to strong bronze tinge, occasionally metallic blue, transscutal articulation along axilla pale yellow. Antenna pale yellow to brownish yellow, basal 3/4 of pedicel slightly infuscate dorsally. Legs predominantly pale yellow, except following parts: fore coxa pale yellow to brown at base, mid coxa infuscate basally to entirely metallic green, hind coxa metallic green with a bronze tinge, tarsomere 4 slightly infuscate, especially at apex, and claws brown. Wings hyaline, with pale yellow tegula and veins (Figure 3a,b).

**Figure 4:**
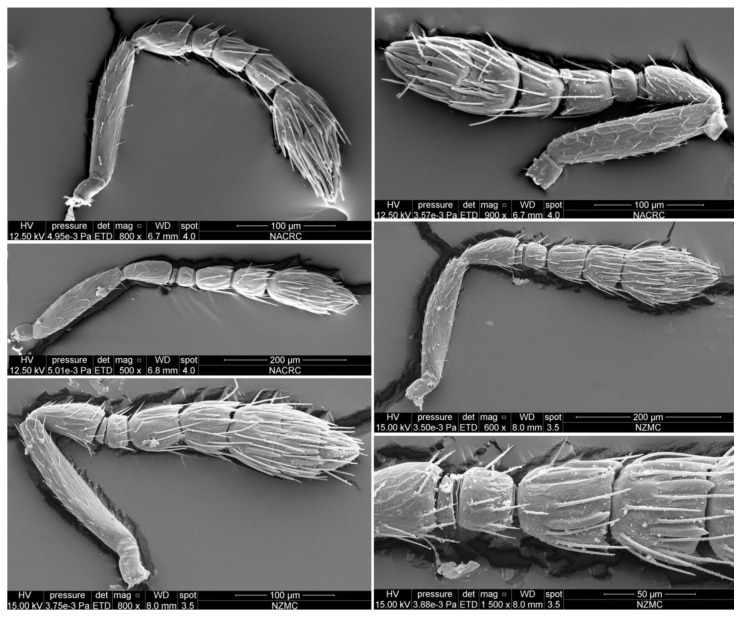
*Dzhanokmenia brevifunis* sp. nov., non-type females, variation in the antenna.

Antenna with 1 transverse anellus, 3 funiculars, and 3 clavomeres (Figure 4); scape without plaque; pedicel distinctly longer than each funicular, usually shorter than and occasionally subequal in length with combined length of F1 and F2; F1 transverse, 1.25–1.55× as broad as long; F2 subquadrate to slightly longer than broad, 1.05–1.45× as long as broad, always longer than F1 and F3; F3 slightly transverse to subquadrate, 0.90–1.25× as broad as long; clava broader than funicle, 0.98–1.15× (without terminal spine) as long as funicle, clavomeres decreasing in length, clavomere 3 truncate apically, with an indistinct terminal spine (Figure 4). F1 with few scattered mushroom-shaped capitate peg sensilla apically, without longitudinal sensilla, remaining flagellomeres with longitudinal sensilla and apically with a circle of scattered, mushroom-shaped capitate peg sensilla (Figure 4).

**Figure 5:**
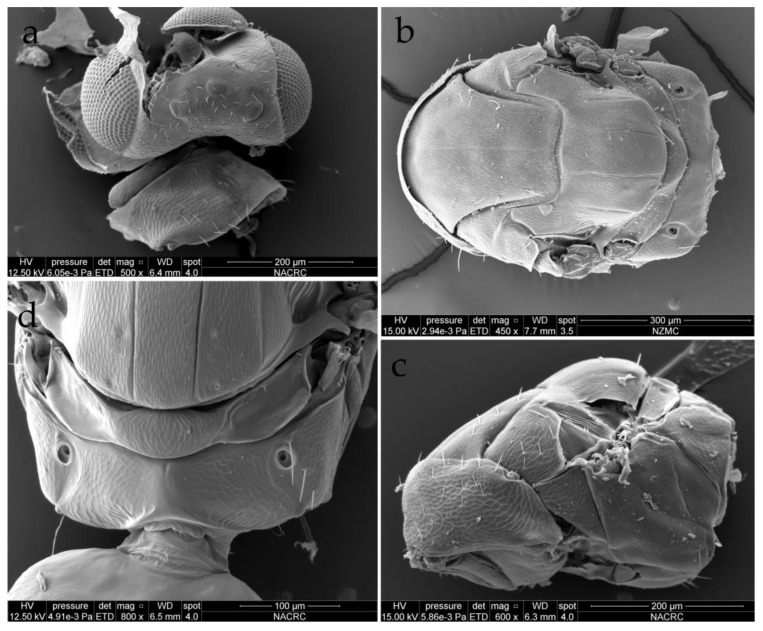
*Dzhanokmenia brevifunis* sp. nov., non-type females. (**a**) Head in dorsal view; (**b**) mesosoma in dorsal view; (**c**) metanotum and propodeum in dorsal view; (**d**) mesosoma in lateral view.

**Figure 6:**
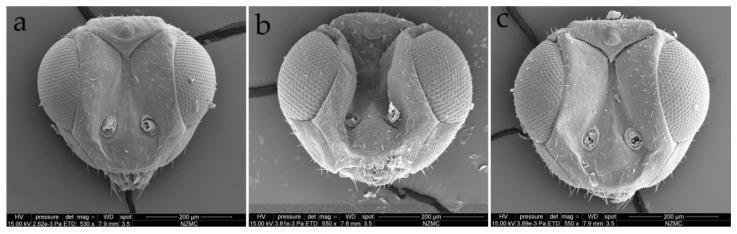
*Dzhanokmenia brevifunis* sp. nov., non-type females. (**a**) Head in front view of slightly collapsed specimen; (**b**) head in front view of specimen with collapsed face along scrobes; (**c**) head in front view of moderately collapsed specimen.

Head slightly broader than mesoscutum and very easily collapsing after death (even if preserved in ethanol). POL about 3× as long as OOL (Figure 5a). Ocelli arranged in an obtuse-angled triangle (Figure 5a). Frons with a broad V-shaped frontofacial suture connecting to ocellar area; upper face with a weak carina between scrobes; head easily collapsing along frontofacial suture, outer margin of scrobes, and outer margin of ocelli (Figure 6a–c). Face with weak reticulation, and even more weak on scrobes. Toruli inserted almost at the same level as lower margin of eyes (Figure 6a).

Vertex, occiput, and upper face with scattered short white setae (scrobal area bare), lower face with sparse longer white setae (Figure 5a and Figure 6a–c). Eyes with short and sparse white setae, height of eye longer than malar space, about 1.8× as long as malar space. Malar space 0.8–0.9× as long as width of mouth opening, with malar sulcus strongly curved (Figure 6b). Anterior margin of clypeus bilobed; mandible bearing 3 teeth, with the length increasing (weak to strong) from the inner to the outer tooth (Figure 6b).

Pronotum strongly sloping and short in dorsal view, neck and collar not delimited, with scattered setae and raised reticulation (Figure 5a,c). Mesoscutum with raised reticulation that is slightly elongate; MLM nearly as long as maximum width, with a well-defined and complete median line, with a row of 2–4 adnotaular setae on each side, symmetric or asymmetric (i.e., with different number or position of setae on each side); axillae shifted strongly forward, with raised reticulation (Figure 5b). Mesoscutellum broader than long, about 1.3× as broad as long, convex in profile, with raised reticulation that is slightly elongate; mesoscutellum with a pair of strong submedian lines and a pair of weak but distinct sublateral lines (Figure 5b), and two pairs of setae, one pair situated slightly below the middle of mesoscutellum and the other pair situated near posterior margin; mesoscutellum with a narrow frenum, almost invisible in dorsal view because of convex mesoscutellum. Dorsellum convex, with reticulation as strong as that on mesoscutellum; lateral panels of metanotum depressed and smooth (Figure 5d).

Propodeum incised medially along anterior and especially posterior margins and thus short medially, 1.1–1.3× as long as median length of dorsellum; propodeum with raised reticulation, slightly weaker than that on dorsellum; median carina on propodeum hard to see but appears to be present due to elevated median area visible when viewed under a microscope; propodeum without plicae, but with curved paraspiracular carinae along inner margin of spiracles; spiracles medially large with entire rim exposed and separated from anterior margin of propodeum by half or more of the diameter of spiracle; callus reticulate, often with 3 short setae, occasionally with 2 setae (Figure 5b,d). Lateral panels of pronotum and prepectus with reticulation as strong as on pronotum, mesepimeron and mesepisternum with weak reticulation, almost smooth; acropleuron smooth; metapleuron reticulate (Figure 5c).

**Figure 7:**
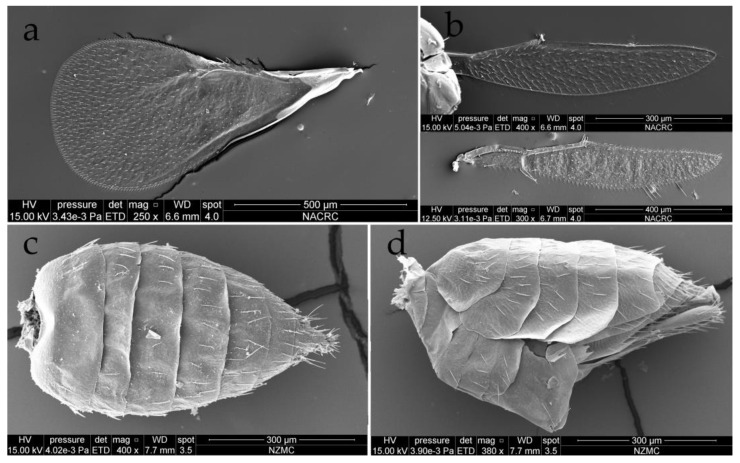
*Dzhanokmenia brevifunis* sp. nov., non-type females. (**a**) Fore wing; (**b**) hind wings; (**c**) gaster in dorsal view; (**d**) gaster in lateral view.

Petiole short and hidden in dorsal view (Figure 3b and Figure 5d). Gaster slender, pointed apically, 1.8–2.0× as long as broad; gastral tergites each with weak raised reticulation; Gt_7_ with 4 cercal setae, subequal in length; hypopygium nearly reaching to 1/2 the length of gaster (Figure 7c,d).

Legs with tarsomere 1 distinctly shorter than the other tarsomeres, tarsomere 2 and tarsomere 3 subequal in length and slightly shorter than tarsomere 4. Fore wing with only MV having long marginal setae, setae on margin of membrane are extremely short, which is challenging to discern even under a microscope, so it looks as if they are missing; fore wing disk without setae below SMV and MV; fore wing disk with short white setae becoming shorter towards base, and basal triangular area before the end of parastigma bare, and speculum is thus large and open below; PMV absent; SMV with 1 seta on dorsal surface (Figure 3a,b and Figure 7a). Hind wing knife-shaped, with the upper margin lacking marginal setae, and the lower margin with soft long white setae that are challenging to discern under a microscope and even in a SEM (Figure 7b).

**Figure 8:**
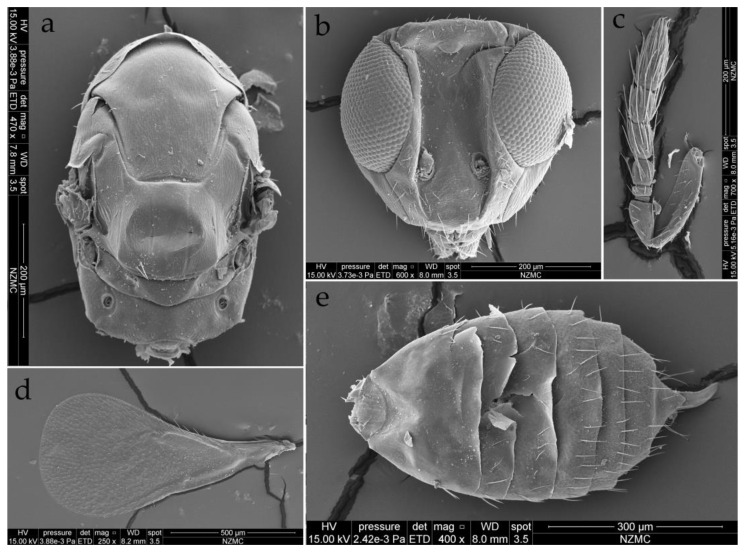
*Dzhanokmenia brevifunis* sp. nov., non-type male. (**a**) Mesosoma in dorsal view; (**b**) Head in front view; (**c**) Antenna; (**d**) Fore wing; (**e**) Gaster in dorsal view.

Male. The males have similar body length to the females and differ from females as follows (Figure 3b,c and Figure 8a–e). Antenna more slender; the median part of scape slightly extending beyond the level of the scape; antenna with funicle 4-segmented; pedicel longer than broad, about 4.5× as long as F1; F1 distinctly transverse, about 2× as broad as long; F2 slightly longer than broad, about 2.5× as long as F1; F3 about 1.4× as long as broad, about 3.6× as long as F1; F4 about 1.2× as long as broad, and nearly as long as F3 (Figure 8c). Middle coxa predominantly metallic green, slightly pale brown apically (Figure 3b,c).

Etymology. From the Latin words ‘brevis’ (meaning ‘short’) and ‘funis’ (from ‘funicle’) (noun in apposition), referring to the transverse F1.

Type material. Holotype ♀, China, Xinjiang, the Desert Plant Garden of Tulufan (42.8542° N, 89.1928° E, −86 m), collected with Malaise Traps, 05-V-2013–15-V-2013, coll. Rui-Xia Liu (IOZCAS, IOZ(E)224671). Paratypes: 2♀2♂, same data as the holotype (IOZCAS, IOZ(E)224672–224675); 6♀, same data as the holotype, except for the collecting dates 25-IV-2013–05-V-2013 (IOZCAS, IOZ(E)224676–224681); 6♀, same data as the holotype, except for the collecting dates 05-V-2023–15-V-2013 (IOZCAS, IOZ(E)224682–224687); 3♀, China, Qinghai, Ge-Ermu, Baiyun Village on the Qinghai Province Road 303 (36.6103° N, 94.8506° E, 2805 m), swept on *Tamarix* sp., 17-VII-2017, coll. Qing-Tao Wu (IOZCAS, IOZ(E)224688–224690); 5♀1♂, China, Xinjiang, A-Letai, Fuhai (47.14604° N, 87.5549° E, 500 m), swept on *Tamarix* sp., coll. Zhi-Liang Wang (IOZCAS, IOZ(E)224691–224696).

Additional material. 6♀ voucher specimens for DNA barcodes, same data as the holotype; 4♀, same data as the holotype, except for the collecting dates 25-IV-2013–05-V-2013; 4♀, same data as the holotype, except for the collecting dates 05-V-2013–15-V-2013; 1♀1♂ on slides, China, Qinghai, Ge-Ermu, Baiyun Village on the Qinghai Province Road 303 (36.6103° N, 94.8506° E, 2805 m), swept on *Tamarix* sp., 17-VII-2017, coll. Qing-Tao Wu; 6♀ voucher specimens for DNA barcoding, China, Qinghai, Ge-Ermu, Baiyun Village on the Qinghai Province Road 303 (36.6103° N, 94.8506° E, 2805 m), swept on *Tamarix* sp., 17-VII-2017, coll. Qing-Tao Wu; 5♀ voucher specimens for DNA barcoding, China, Xinjiang, A-Letai, Fuhai (47.14604° N, 87.5549° E, 500 m), swept on *Tamarix* sp., coll. Zhi-Liang Wang; 16♀ preserved in 95%–100% ethanol, same data as holotype; 6♀, preserved in 95%–100% ethanol, same data as the holotype, except for the collecting dates 25-IV-2013–05-V-2013; 16♀, preserved in 95–100% ethanol, same data as the holotype, except for the collecting dates 05-V-2013–15-V-2013.

Host information. The holotype and some of the paratypes were collected by sweeping shrubs of *Tamarix* sp. in Xinjiang and Qinghai, China.

Distribution. Palearctic region (China: Qinghai, Xinjiang). The records of this species in semi-arid areas from both low and high altitudes suggest a potential distribution range spanning from about −100 to 2800 m above sea level.

Remarks. The specimens collected on the Tibetan Plateau at high altitudes have a larger body size than those collected in the Turpan Basin at low altitudes. *Dzhanokmenia brevifunis* shows variation in the coloration of the coxae on the fore and middle legs, as well as in the relative length of each funicular. In females, the fore coxa typically appears pale yellow, with a dark brown coloration that spreads to varying degrees, but never entirely covers the coxa or has a metallic green tint. However, the dark brown coloration of the middle coxa in females may extend from the base to the entire surface and sometimes have a metallic green tinge, similar to the color of the body. In addition, differences in the relative length of each funicular can be quite confusing due to their wide range of variation. However, F1 is consistently transverse and never subquadrate or longer than broad. In contrast, the fore coxa in males is typically pale yellow but may also have a dark brown color or even be entirely dark brown or metallic green. The relative length of each funicular appears to be relatively consistent compared to females. The undetectable variation in the male antenna may be attributed to the limited number of male individuals within a brood sample. The confirmation of these morphological variations was corroborated by the results of molecular species delimitation. For instance, the species represented by CHX_DZH_893 exhibits a fore coxa that is entirely metallic green and a subquadrate F1. Additionally, the species represented by CHX_DZH_898, which was also recognized from specimens swept on *Tamarix* sp., has antennae similar to *D. brevifunis* and entirely yellow fore and middle coxae but possesses a distinct gaster with yellow Gt_1–2_.

The species referred to as “*D.* cf. *brevifunis*” in Table 1 and Figure 1 (as well as in Figures S1 and S2) is a smaller species with a body length of about 0.8 mm compared to *D. brevifunis*. It can be distinguished from *D. brevifunis* by the combination of the following characters: all coxae entirely metallic green; callus with 2 setae. Sometimes the antenna of *D.* cf. *brevifunis* can be distinguished from that of *D. brevifunis*, with transverse F1 being longer than 1/2 length of F2 and F2 being subquadrate and subequal to F3 in length. However, this species also shows variation in the relative length of each funicular, which complicates the comparison of interspecific variation between these two species, especially when dealing with a limited number of individuals of *D.* cf. *brevifunis*. Despite few distinctions, we treated *D.* cf. *brevifunis* as a species distinct from *D. brevifunis*. In *D. brevifunis*, the fore coxa is never entirely metallic green, and the callus typically has 3 setae; if the callus has 2 setae, then F2 and F3 are both slightly longer than broad.

Morphologically, *Dzhanokmenia brevifunis* appears to be closely related to species of this genus that have a completely metallic gaster. Among these species, *D. brevifunis* appears to be most closely related to *D. antonovae*, as confirmed by the ML trees (Figures S1 and S2). The clearly transverse F1 distinguishes *D. brevifunis* from these species within *Dzhanokmenia*. However, *D. karamayica*, another Chinese species with metallic gaster, was clustered with the other three species with partially yellow gaster (*D. gobia*, *D. muleica,* and *D.* cf. *sugonjaevi*) rather than with *D. brevifunis* in the ML trees (Figures S1 and S2). Compared to these two clusters, the cluster containing *D. antonovae* and *D. brevifunis* has F1 transverse or at most subquadrate, while the other cluster containing *D. karamayica* has F1 longer than broad.

## 4. Discussion

In this study, we describe a new species of *Dzhanokmenia* distributed in Xinjiang and Qinghai, China, and it represents the first record of the genus from the semi-arid region of the Tibetan Plateau in Qinghai Province, China. A preliminary DNA barcode library for *Dzhanokmenia* was also constructed, revealing the potential species diversity around *D. brevifunis* by delimiting variation. Although the topology of the ML tree varies depending on the taxa or DNA regions included, the phylogenetic results nevertheless indicate a potential close relationship between *Dzhanokmenia* and *Baryscapus*, as well as to other genera associated with *Tetrastichus*, such as *Chaenotetrastichus* and *Oomyzus*.

One of the major challenges in species delimitations based on morphology arises from the complexities involved in discerning intra- and interspecific variation in morphological characters. This study unveiled that *Dzhanokmenia* species can manifest two extreme conditions. In one scenario, they display minor morphological variation but significant interspecific genetic differences (e.g., *D. brevifunis* and *D.* cf. *brevifunis*). Under this condition, specimens associated with a cluster of plant hosts may potentially represent distinct species. In the other scenario, they exhibit substantial morphological diversity yet demonstrate limited intraspecific genetic variation (e.g., the relative length of each funicular in *D. brevifunis*). In this case, there is a risk of mistakenly dividing one species into multiple entities. Therefore, a large number of specimens are essential for discussing intra- and interspecific morphological variation, which helps prevent the erroneous inclusion of closely related species under one classification or the excessive division of species.

The DNA barcoding analysis conducted in this study revealed a species complex containing at least two species. For specimens swept from adjacent clusters of *Tamarix* sp. (Tamaricaceae) and collected at a specific site with a Malaise trap, each genetically contained *D. brevifunis* and another distinct but occasional species. This occasional species, represented by *D.* cf. *brevifunis* (see Table 1 and Figure 2), remains undescribed due to an insufficient amount of material and was, therefore, not described here. Further extensive surveys of *Dzhanokmenia* in semi-arid regions and the acquisition of adequate material are needed to determine whether the currently observed variation in *D.* cf. *brevifunis* in a limited number of specimens can be classified as intra- or interspecific.

In addition, the other *Dzhanokmenia* species examined in this study are valuable for verifying the significance of variation in the relative length of each funicular (especially F1), the presence of a median line on the MLM, and the color of legs and gaster, as key characters for species delimitation within *Dzhanokmenia*. It is noteworthy that the range of variation in delimiting *Dzhanokmenia* species can vary between species, potentially increasing the challenges of delimitation in the absence of molecular data. The two similar species, *D. gobica* and *D. muleica*, which are primarily distinguished by differences in vertex and gaster [14], were not differentiated in the ML trees based on the 28S gene (Figures S1 and S2). In addition, *D. karamayica*, with an entirely metallic green gaster, is clustered with *D. gobica* and *D. muleica,* which have a predominantly yellow gaster. Future work is needed to determine whether the color variation in the gaster or vertex can be considered intraspecific in these species. In contrast, the yellow Gt_1–2_ directs the species represented by CHX_DZH_898 away from *D. brevifunis* (Figure 1 and Figure 2), while the variation in the above-mentioned characters fall within the range of *D. brevifunis*. All species have a slender F1 (longer than broad) in one cluster and a transverse F1 (at most subquadrate) in the other cluster in all three ML trees, suggesting that the antenna may take precedence over the other three characters mentioned above in grouping *Dzhanokmenia* species.

The topology of the branch containing *Dzhanokmenia* in the ML tree based on MatrixII (Figure S2) shows slight differences compared to that based on MatrixI (Figure S1). In the former scenario, the genus *Dzhanokmenia* is grouped in one cluster, whereas in the latter, it is divided into two clusters interspersed with four other genera: *Baryscapus*, *Chaenotetrastichus*, *Oomyzus*, and *Tetrastichus*. The ML tree based on MatrixIII, concatenating COI and 28S, shows a similar topology to the tree based on MatrixI. The deviations in the topologies of the ML trees based on different datasets show that the trees are not robust. Nevertheless, all scenarios suggest possible close relationships of these genera associated with *Tetrastichus* included in the ML analyses. Although confirming the monophyly of *Dzhanokmenia* or *Baryscapus* requires a well-resolved phylogeny of these genera, these trees provide clues for understanding the relationships among these genera. Further research on *Dzhanokmenia* and *Baryscapus* will be impeded without a comprehension of their relationships.

This study provides a preliminary DNA barcode library for the genus *Dzhanokmenia* that will serve as a valuable resource for the identification of both intra- and interspecific variation. It is anticipated that this library will aid in future efforts in species discovery and description within the genus. However, the construction of a more comprehensive DNA barcode library for *Dzhanokmenia* remains essential and requires urgent attention. Analyses of the limited DNA data suggest a potentially close relationship between *Dzhanokmenia* and other genera related to *Tetrastichus*, beyond *Baryscapus*. Nonetheless, the current dataset lacks the robustness necessary to definitively establish the monophyly of these genera. As Cao et al. [25] emphasized, a more comprehensive dataset that includes a broader range of taxa and incorporates multiple lines of evidence is necessary to elucidate the phylogenetic relationships of these genera.

## Data Availability

DNA data generated in this study are available on GenBase under accession numbers (see Table 1) and other public DNA data downloaded from GenBank are available under corresponding accession numbers mentioned in Table S1. Other data used in this study are deposited at the Institute of Zoology, Chinese Academy of Sciences, Beijing, China.

## Supplementary Materials

The following supporting information can be downloaded at: https://www.mdpi.com/article/10.3390/insects15060406/s1, Figure S1: An ML tree based on MatrixI of 28S D2 sequences, with species from the tribe Cirrospilini as outgroups; Figure S2: An ML tree based on MatrixII of 28S D2 sequences, with *Styotrichia pixoxylona* as the outgroup; Table S1: List of public sequences used in the ML analyses with accession numbers from GenBank.
