# Supplementary material for: One Step beyond Species Description: Unveiling a Fine-Scale Diversity within the Genus Dzhanokmenia Kostjukov (Hymenoptera: Eulophidae)
Source: Insects. 2024 Jun 1;15(6):406. doi: 10.3390/insects15060406 (PMC11203707; doi:10.3390/insects15060406)
Supplement: Supplementary file 1 [file insects-15-00406-s001.zip › insects-3018912-supplementary.pdf]

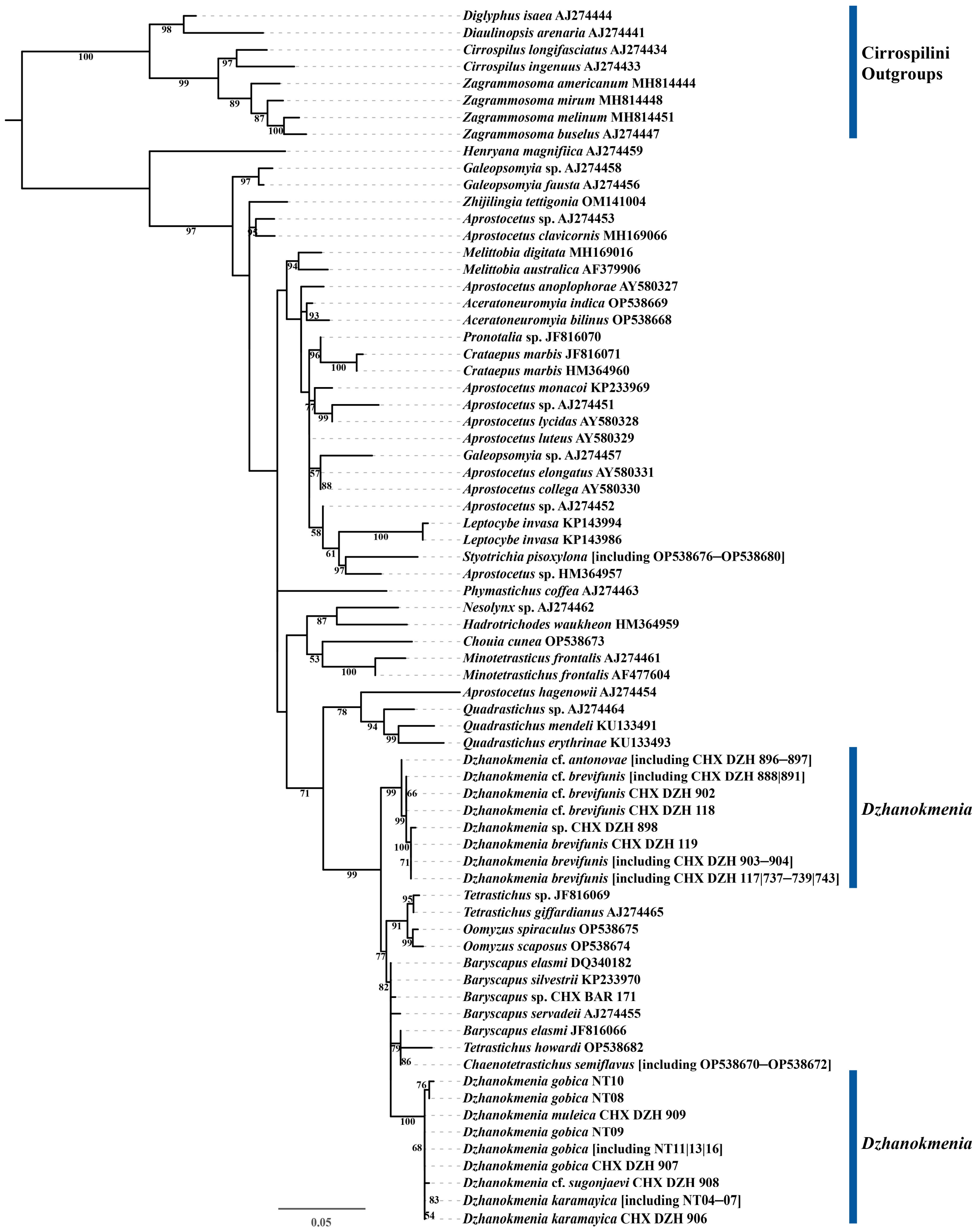

Figure S1. A ML tree based on MatrixI of 28S D2 sequences, with species from the tribe Cirrospilini as outgroups. Bootstrap values are shown beside to the nodes with values greater than 50. Blue vertical bars indicate the outgroups or the *Dzhanokmenia* species.

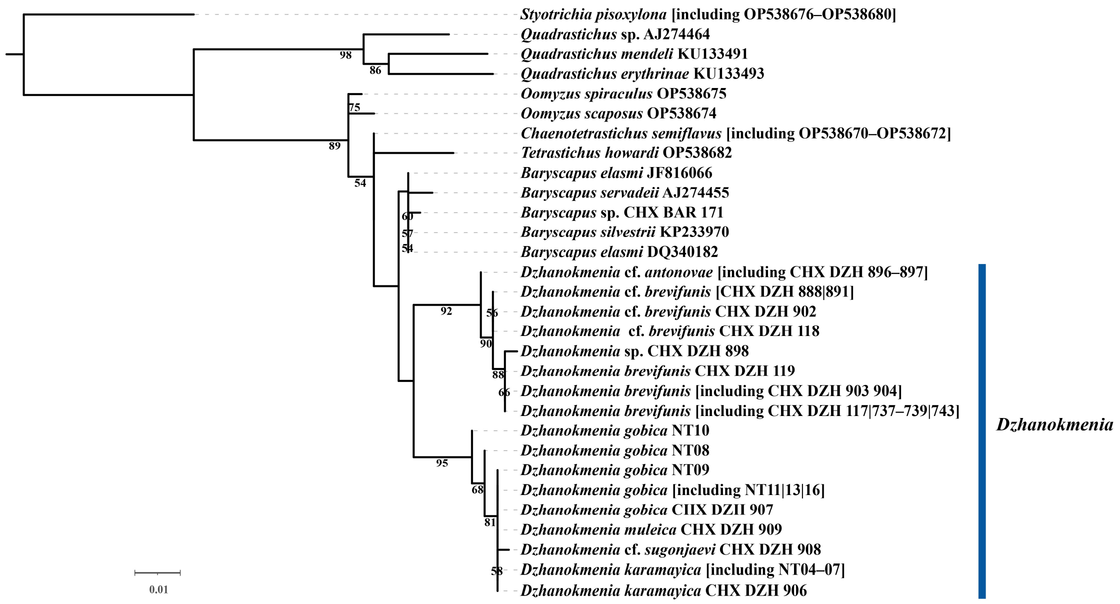

Figure S2. A ML tree based on MatrixII of 28S D2 sequences, with *Styotrichia pixoxylona* as outgroup. Bootstrap values are shown beside to the nodes with values greater than 50. Blue vertical bars indicate the *Dzhanokmenia* species.

Table S1. List of public sequences used in the ML analyses with accession numbers from GenBank.

Terminal in the tree	Gene	Species	Sample voucher	Accession Number	Locality Collected in
Tetrastichus howardi CHX_TET_054	28S D2	*Tetrastichus howardi*	CHX_TET_054	OP538682	Brazil, MG, Sete Lagoas
Chouia cunea OP538673	28S D2	*Chouioia cunea*	CHX_CHO_139	OP538673	China, ShanDong Province
Styotrichia pisoxylona OP538676	28S D2	*Styotrichia pisoxylona*	CHX_STY_749	OP538676	China, JiangXi Province
Styotrichia pisoxylona OP538677	28S D2	*Styotrichia pisoxylona*	CHX_STY_749	OP538677	China, JiangXi Province
Styotrichia pisoxylona OP538678	28S D2	*Styotrichia pisoxylona*	CHX_STY_749	OP538678	China, JiangXi Province
Styotrichia pisoxylona OP538679	28S D2	*Styotrichia pisoxylona*	CHX_STY_749	OP538679	China, JiangXi Province
Styotrichia pisoxylona OP538680	28S D2	*Styotrichia pisoxylona*	CHX_STY_749	OP538680	China, JiangXi Province
Styotrichia pisoxylona OP538681	28S D2	*Styotrichia pisoxylona*	CHX_STY_749	OP538681	China, JiangXi Province
Chaenotetrastichus semiflavus OP538670	28S D2	*Chaenotetrastichus semiflavus*	CHX_CHA_791	OP538670	China, JiangXi Province
Chaenotetrastichus semiflavus OP538671	28S D2	*Chaenotetrastichus semiflavus*	CHX_CHA_791	OP538671	China, JiangXi Province
Chaenotetrastichus semiflavus OP538672	28S D2	*Chaenotetrastichus semiflavus*	CHX_CHA_791	OP538672	China, JiangXi Province
Chaenotetrastichus semiflavus OP538674	28S D2	*Oomyzus scaposus*	Oomyzus_scaposus_F1	OP538674	China, JiangSu Province
Aceratoneuromyia bilinus OP538668	28S D2	*Aceratoneuromyia bilinus*	CHX_ACE_787	OP538668	China, Yunnan, Yingjiang
Aceratoneuromyia indica OP538669	28S D2	*Aceratoneuromyia indica*	CHX_ACE_833	OP538669	China, Fujian, Fuzhou
Oomyzus spiraculus OP538675	28S D2	*Oomyzus spiraculus*	Oomyzus_spiraculus_F1	OP538675	China, Jiangsu, Nanjing
Zhijilingia tettigonia OM141004	28S D2	*Zhijilingia tettigonia*		OM141004	
Melittobia australica AF379906	28S D2	*Melittobia australica*		AF379906	
Aprostocetus clavicornis MH169066	28S D2	*Aprostocetus clavicornis*		MH169066	
Melittobia digitata MH169016	28S D2	*Melittobia digitata*		MH169016	
Quadrastichus erythrinae KU133493	28S D2	*Quadrastichus erythrinae*		KU133493	
Quadrastichus mendeli KU133491	28S D2	*Quadrastichus mendeli*		KU133491	
Baryscapus silvestrii KP233970	28S D2	*Baryscapus silvestrii*		KP233970	
Aprostocetus monacoi KP233969	28S D2	*Aprostocetus monacoi*		KP233969	
Leptocybe invasa KP143994	28S D2	*Leptocybe invasa*		KP143994	
Leptocybe invasa KP143986	28S D2	*Leptocybe invasa*		KP143986	

**Terminal in the tree**	**Gene**	**Species**	**Sample voucher**	**Accession Number**	**Locality Collected in**
Crataepus marbis JF816071	28S D2	*Crataepus marbis*		JF816071	
Pronotalia sp. JF816070	28S D2	*Pronotalia* sp.		JF816070	
Tetrastichus sp. JF816069	28S D2	*Tetrastichus* sp.		JF816069	
Baryscapus elasmi JF816066	28S D2	*Baryscapus elasmi*		JF816066	
Crataepus marbis HM364960	28S D2	*Crataepus marbis*		HM364960	
Hadrotrichodes waukheon HM364959	28S D2	*Hadrotrichodes waukheon*		HM364959	
Aprostocetus sp. HM364957	28S D2	*Aprostocetus* sp.		HM364957	
Minotetrastichus frontalis AF477604	28S D2	*Minotetrastichus frontalis*		AF477604	
Aprostocetus elongatus AY580331	28S D2	*Aprostocetus elongatus*		AY580331	
Aprostocetus collega AY580330	28S D2	*Aprostocetus collega*		AY580330	
Aprostocetus luteus AY580329	28S D2	*Aprostocetus luteus*		AY580329	
Aprostocetus lycidas AY580328	28S D2	*Aprostocetus lycidas*		AY580328	
Aprostocetus anoplophorae AY580327	28S D2	*Aprostocetus anoplophorae*		AY580327	
Baryscapus elasmi DQ340182	28S D2	*Baryscapus elasmi*		DQ340182	
Galeopsomyia sp. AJ274458	28S D2	*Galeopsomyia* sp.		AJ274458	
Galeopsomyia sp. AJ274457	28S D2	*Galeopsomyia* sp.		AJ274457	
Aprostocetus sp. AJ274453	28S D2	*Aprostocetus* sp.		AJ274453	
Aprostocetus sp. AJ274452	28S D2	*Aprostocetus* sp.		AJ274452	
Aprostocetus sp. AJ274451	28S D2	*Aprostocetus* sp.		AJ274451	
Quadrastichus sp. AJ274464	28S D2	*Quadrastichus* sp.		AJ274464	
Nesolynx sp. AJ274462	28S D2	*Nesolynx* sp.		AJ274462	
Tetrastichus giffardianus AJ274465	28S D2	*Tetrastichus giffardianus*		AJ274465	
Phymastichus coffea AJ274463	28S D2	*Phymastichus coffea*		AJ274463	
Minotetrasticus frontalis AJ274461	28S D2	*Minotetrasticus frontalis*		AJ274461	
Henryana magnifiica AJ274459	28S D2	*Henryana magnifica*		AJ274459	
Galeopsomyia fausta AJ274456	28S D2	*Galeopsomyia fausta*		AJ274456	

**Terminal in the tree**	**Gene**	**Species**	**Sample voucher**	**Accession Number**	**Locality Collected in**
Baryscapus servadeii AJ274455	28S D2	*Baryscapus servadeii*		AJ274455	
Aprostocetus hagenowii AJ274454	28S D2	*Aprostocetus hagenowii*		AJ274454	
Zagrammosoma buselus AJ274447	28S D2	*Zagrammosoma buselus*		AJ274447	
Diglyphus isaea AJ274444	28S D2	*Diglyphus isaea*		AJ274444	
Diaulinopsis arenaria AJ274441	28S D2	*Diaulinopsis arenaria*		AJ274441	
Cirrospilus longifasciatus AJ274434	28S D2	*Cirrospilus longifasciatus*		AJ274434	
Cirrospilus ingenuus AJ274433	28S D2	*Cirrospilus ingenuus*		AJ274433	
Zagrammosoma melinum MH814451	28S D2	*Zagrammosoma melinum*		MH814451	
Zagrammosoma mirum MH814448	28S D2	*Zagrammosoma mirum*		MH814448	
Zagrammosoma americanum MH814444	28S D2	*Zagrammosoma americanum*		MH814444	
Chaenotetrastichus semiflavus CHX791	COI	*Chaenotetrastichus semiflavus*	CHX_CHA_791	OP536210	China, JiangXi Province
Chaenotetrastichus semiflavus CHX792	COI	*Chaenotetrastichus semiflavus*	CHX_CHA_791	OP536211	China, JiangXi Province
Chaenotetrastichus semiflavus CHX793	COI	*Chaenotetrastichus semiflavus*	CHX_CHA_791	OP536212	China, JiangXi Province
Styotrichia pisoxylona CHX749	COI	*Styotrichia pisoxylona*	CHX_STY_749	OP536213	China, JiangXi Province
Styotrichia pisoxylona CHX750	COI	*Styotrichia pisoxylona*	CHX_STY_750	OP536214	China, JiangXi Province
Styotrichia pisoxylona CHX751	COI	*Styotrichia pisoxylona*	CHX_STY_750	OP536215	China, JiangXi Province
Styotrichia pisoxylona CHX752	COI	*Styotrichia pisoxylona*	CHX_STY_750	OP536216	China, JiangXi Province
Styotrichia pisoxylona CHX753	COI	*Styotrichia pisoxylona*	CHX_STY_750	OP536217	China, JiangXi Province
Styotrichia pisoxylona CHX754	COI	*Styotrichia pisoxylona*	CHX_STY_750	OP536218	China, JiangXi Province
Oomyzus scaposus F1	COI	*Oomyzus scaposus*	Oomyzus_scaposus_F1	MT259797	China, JiangSu Province
Oomyzus spiraculus F2	COI	*Oomyzus spiraculus*	Oomyzus_spiraculus_F2	MT259806	China, JiangSu Province
